# Organosolv-Water Cosolvent Phase Separation on Cellulose and its Influence on the Physical Deconstruction of Cellulose: A Molecular Dynamics Analysis

**DOI:** 10.1038/s41598-017-15048-7

**Published:** 2017-11-03

**Authors:** Micholas Dean Smith, Xiaolin Cheng, Loukas Petridis, Barmak Mostofian, Jeremy C. Smith

**Affiliations:** 1 Center for Molecular Biophysics, University of Tennessee/Oak Ridge National Laboratory, Oak Ridge, TN 37830 USA; 2 Department of Biochemistry and Cellular and Molecular Biology, University of Tennessee, Knoxville, TN 37996 USA; 3 Oak Ridge National Laboratory, Oak Ridge, TN 37830 USA

## Abstract

Deconstruction of cellulose is crucial for the chemical conversion of lignocellulose into fuel/bioproduct precursors. Recently, a water-organosolv cosolvent system (THF-water) has been shown to both phase-separate on cellulose surfaces and partially deconstruct Avicel  (cellulose) in the absence of acid. Here we employ molecular dynamics simulations to determine whether other common water-organosolv cosolvent systems (acetone, ethanol, and γ-valerolactone) exhibit phase separation at cellulose surface and whether this alters a purely physical cellulose dissociation pathway. Despite finding varied degrees of phase-separation of organosolv on cellulose surfaces, physical dissociation is not enhanced. Interestingly, however, the total amount the median water-cellulose contact lifetimes increases for the cosolvent systems in the order of THF > acetone > ethanol > γ-valerolactone. Together our results indicate two points: a purely physical process for deconstruction of cellulose is unlikely for these cosolvents, and in THF-water, unlike γ-valerolactone- (and some concentrations of acetone and ethanol) water cosolvents, a significant fraction of surface water is slowed. This slowing may be of importance in enhancing chemical deconstruction of cellulose, as it permits an increase in potential THF-water-cellulose reactions, even while the amount of water near cellulose is decreased.

## Introduction

Organic solvents are used for the pretreatment of lignocellulosic biomass to enhance the conversion of cellulose into fermentable sugars^1–3^. Recently, novel water-organosolv pretreatments have demonstrated greatly enhanced sugar yields^4–15^. The presumed mechanism for the increased yields of fermentable sugars is that pretreatments disrupt the interactions of the plant cell-wall polymers: lignin, crystalline cellulose fibres, and hemicellulose^16^; these disruptions then result in a variety of structural changes - for example increasing mesoscale porosity, reducing lignin content, and/or altering cellulose crystallinity^16–19^. The key consequence of these changes is that more cellulose is exposed to solvents for enzymatic or chemical upgrading. There is considerable interest in understanding how cosolvents can lead to such a dramatic increase in pretreatment efficiency, and if the potentially complex phase behaviour of cosolvents at biomass-solvent interfaces plays a significant role.

Several studies focusing on tetrahydrofuran-water (THF-water) and γ-valerolactone-water (GVL-water) cosolvent systems have demonstrated that, apart from solubilizing lignin and hemicellulose, crystalline cellulose can itself also be partially solubilized/decrystallized by these cosolvents^5,8–10,20–23^. Furthermore, experimental examination of the use of THF-water cosolvents on Avicel has demonstrated that the cosolvent mixture facilitates cellulose hydrolysis^21^. The mechanism behind this partial solubilization/decrystallization of cellulose under these conditions is still not entirely clear; however, for the case of THF-water, it has been suggested that the previously reported phase-separation of THF and water upon the cellulose surface may be responsible^21^.

The influence of organosolv-water cosolvent pretreatments on lignocellulosic biomass is likely multifaceted, possibly involving both chemical and physical processes that enhance sugar yields. Among the outstanding questions are what type of behaviour occurs at the cosolvent-cellulose interface, whether interfacial interactions enhance or modify the physical deconstruction of cellulose, and whether it is physical or chemical dissociation that becomes more energetically favourable in water-organosolv cosolvents. We address these questions by focusing on solvation and a hypothetical physical dissociation of a cellulose strand from cellulose fibres in four different cosolvent mixtures (THF, acetone, GVL, and ethanol at 1:1 v/v, 1:2 v/v and 7:10 v/v ratios with water). The use of ethanol and acetone as other cosolvents to be included in this study is motivated by their historical use in biomass pretreatment^1,3,12^. For comparison, we also calculate the dissociation/peeling free-energies in purely aqueous and salt-water (0.2 M, 0.4 M, 0.6 M) environments. We use classical all-atom molecular dynamics simulations to probe the cellulose-cosolvent interface in these cosolvent mixtures, focusing on preferential solvation properties. We also extract the free-energy barrier of peeling a central, top-layer, cellulose chain from a fibre to quantify how interfacial differences affect a purely physical cellulose deconstruction.

## Computational Methodology

All-atom molecular dynamics simulations coupled with umbrella-sampling were used to characterize cosolvent behaviour on the surface of cellulose and to obtain potential of mean force (PMF) profiles of dissociating/peeling an imperfect (19 glucose units in length) central top layer strand from a (20-glucose unit) cellulose fibre’s surface while solvated in acetone, ethanol, GVL, and THF cosolvents. The imperfection is included to avoid edge effects and to better represent real biomass as real biomass is unlikely to be defect free. Each organic solvent was examined at four different concentrations, pure, 1:1 v/v, 7:10 v/v, and 1:2 v/v organosolv:water, and compared to PMFs obtained under pure water and salt-water (0.2 M, 0.4 M, 0.6 M NaCl) conditions.

Simulations, using the GROMACS 5.1 software suite^24^, were performed in the NPT and NVT ensembles, with P~1 bar and T = 303 K. Temperatures were controlled using the V-Rescale algorithm^25^ and pressure was controlled using the Berendsen barostat^26^. To allow for an integration time step of 2fs for all simulations, all bonds were constrained using the P-LINCS algorithm^27,28^. Force-field parameters for THF, acetone, ethanol, cellulose, water, and NaCl were obtained from the CHARMM36 force-field^29^. GVL parameters were taken from the CHARMM general force-field^30–32^ (obtained from the online CHARMM-GUI webpage^33,34^).

**Figure 1:**
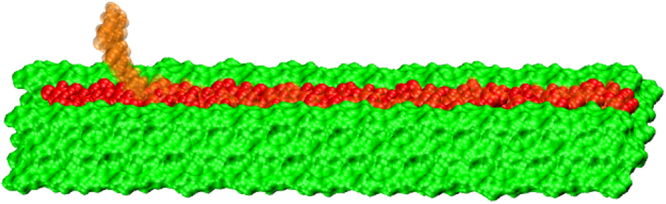
Initial half-sheet-cellulose fibre structure. The red chain is the initial position of the pulled chain and the transparent orange is the final pulled position.

Simulations of each solvent-cellulose system were performed following a four-step process: equilibration, non-equilibrium pulling/umbrella window generation, independent window relaxation, and independent window production simulations. Equilibration was performed in two steps, the first being a standard energy minimization to remove clashes between atoms that may have been generated during the solvation of the model. The minimization was performed using the steepest-descent algorithm for up to 25000 steps or until the convergence tolerance (whichever was reached first) of 23.9 kcal mol^−1^ nm^−1^ (or 17.93 kcal mol^−1^ nm^−1^ in the case of GVL) was reached. Following the minimization procedure, a short (10 ns) NPT (P = 1 bar, T = 303 K) simulation was performed to relax the system and allow the pressure to reach ~1 bar. An example system is presented in Fig. 1. For computational efficiency, only three layers, with three chains on the top layer, followed by four and then five on the bottom layer, with each chain composed of 20 glucose-unit chains (excluding the defect pulled-centre chain, which, as noted above, was composed of 19 glucose units) of the cellulose fibre were simulated within a rectangular periodic box.

The interface between the (co)solvent environment and the cellulose was characterized by calculating the amount of hydrogen bonding between the (co)solvent components and the pulled cellulose strand, the radial distribution functions of the (co)solvent components relative to the pulled strand, the 2D density profile of the cosolvent components on the cellulose surface, the cumulative distribution of sub-5ns lifetimes of individual water molecules on the cellulose surface (i.e. within 0.5 nm of the peeling surface), and the distribution of the number of water molecules on the cellulose surface. For the calculation of hydrogen bonds, a length cut-off criterion (donor-acceptor distance) of 0.3 nm with an angle cut-off of 20° was used. The interactions between cosolvent components were also characterized by calculating the water-organosolv, water-water, and organosolv-organosolv radial distribution functions and converted to cosolvent interaction virials (by performing the standard integration^35,36^). Radial distribution functions, hydrogen bonds, 2D density profiles, and water lifetimes were computed using the built-in analysis tools of the GROMACS simulation suite^24^.

Non-equilibrium pulling/umbrella window generation simulations were driven with a constant pulling rate of 0.02 nm ps^−1^ and a spring constant of 298.8 kcal mol^−1^ nm^−2^ attached to the reducing end pyranose ring of the central top-layer chain. The non-equilibrium simulations were performed for 250 ps (with an integration time-step of 2fs) in the NVT ensemble with T = 303 K. From the non-equilibrium pulling simulations, 11 windows were selected for umbrella sampling along the reaction coordinate of the Z-distance of the pulled-ring from the centre of mass of the fibre. It should be noted that alternative reaction coordinates (native contacts) for estimating the decrystallization energy have been utilised by other groups^37^; however, we used the reaction coordinate noted in ref.^38^, as it allows us to focus on stepwise glucose detachments.

The umbrella windows for this study were located along the reaction coordinate at the Z-distances: 0.5 nm (corresponding to no detachment), 0.65 nm, 0.75 nm (corresponding to the full detachment of ~1 glucose unit), 0.85 nm, 1 nm, 1.05 nm (corresponding to the full detachment of ~1 cellobiose unit), 1.15 nm, 1.25 nm, and 1.4 nm. Position restraints were then applied on all cellulose atoms and another round of solvent relaxation simulation (10 ns in length) for each window was performed. Finally, umbrella sampling simulations (independent simulations in each window) were performed for 30 ns at T = 303 K, with the same harmonic potential as used to generate the simulation windows applied to the pulled end-sugar and position restraints applied to the remainder of the fibre. Post-processing of the umbrella simulations to calculate the dissociation PMF profiles was performed using the GROMACS implementation of the weighted-histogram analysis method (WHAM)^39,40^, using the last 20 ns of each production umbrella window trajectory, with 50 bins. Error estimates for the WHAM analysis were obtained from 25 bootstraps.

Due to the size of the data produced (over 46 Terabytes) for this study, trajectories and run input files are only available upon request to the authors.

## Results and Discussion

**Figure 2:**
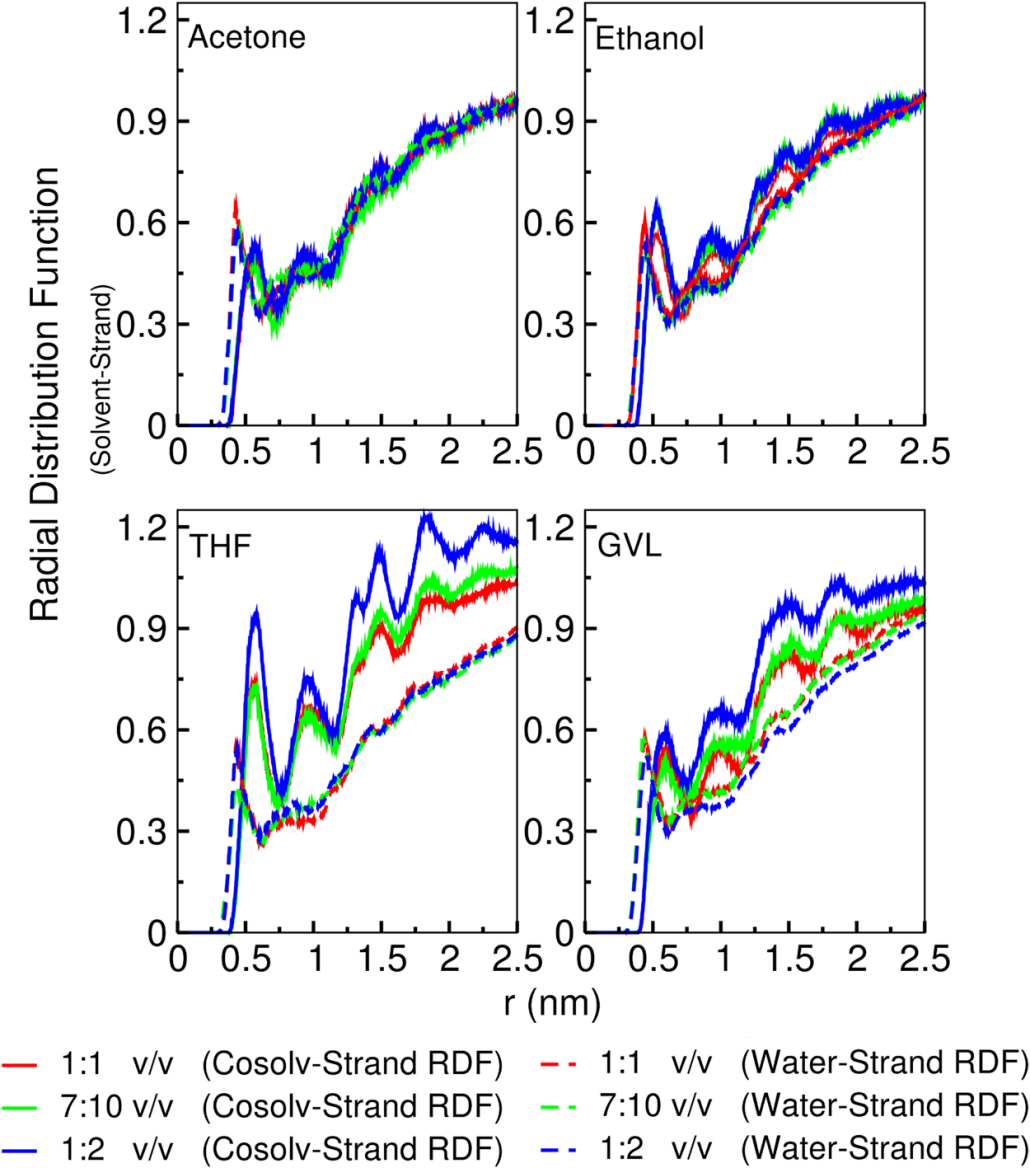
Radial distribution functions of the cosolvent system components with respect to the centre strand of the top layer of the cellulose fibre. Solid colours correspond to the organic solvents and the dashed lines correspond to water. Radial distribution functions are from the first umbrella window (the end of centre strand is not displaced from the fibre surface).

**Figure 3:**
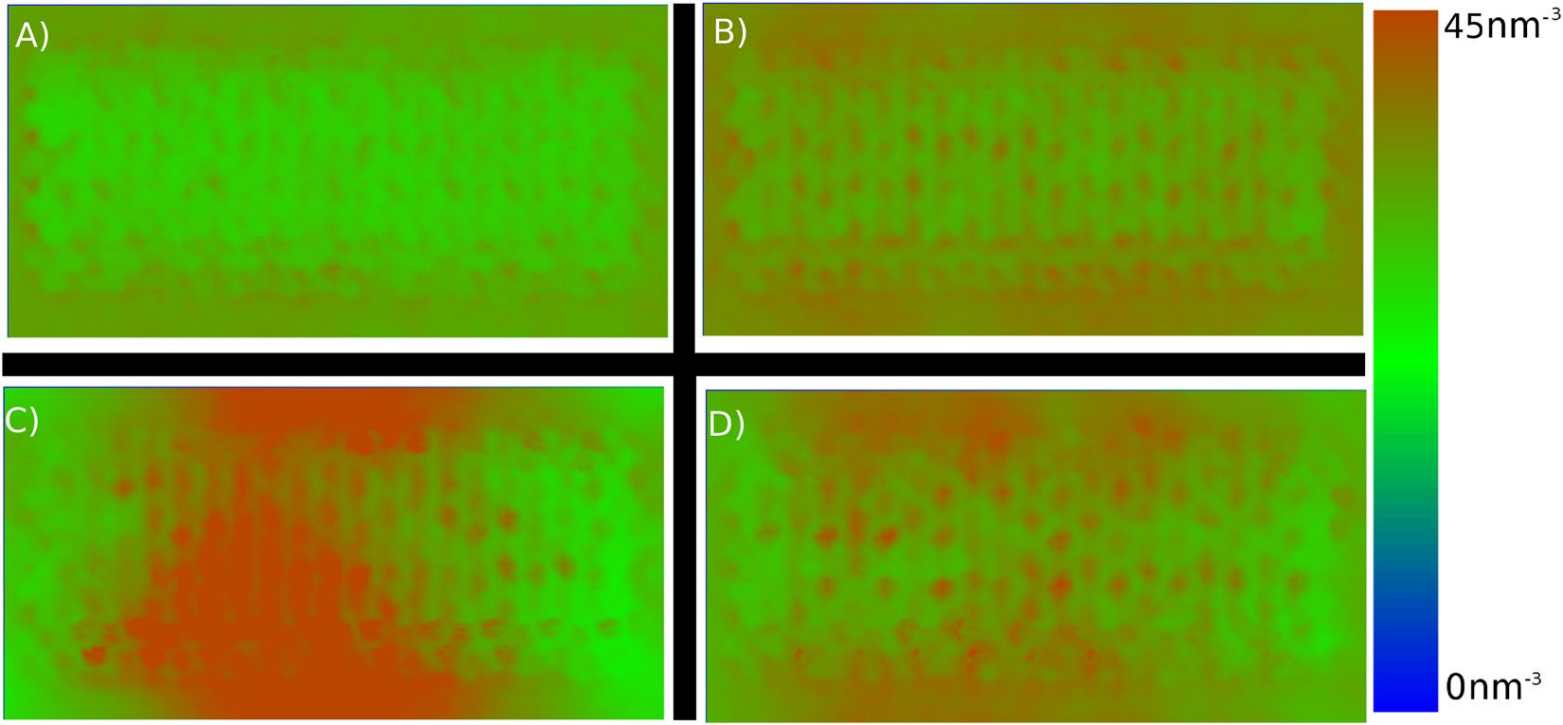
2D Density Profiles of organosolvs at 7:10 v/v ratio on the cellulose surface. (**A**) Acetone, (**B**) Ethanol, (**C**) THF, & (**D**) GVL. A complete comparison of all concentrations is provided in ESI Fig. 1.

**Figure 4:**
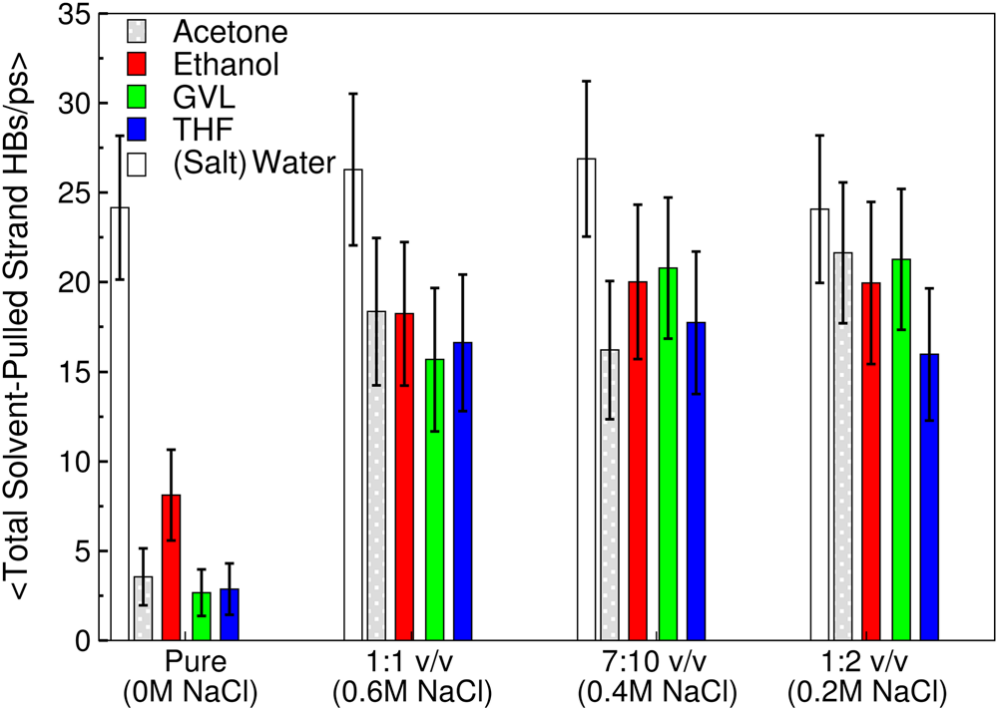
Average number of all solvent (total organosolv and water) hydrogen-bonds with the pulled strand from the first umbrella window. Error-bars are standard error of the mean.

**Figure 5:**
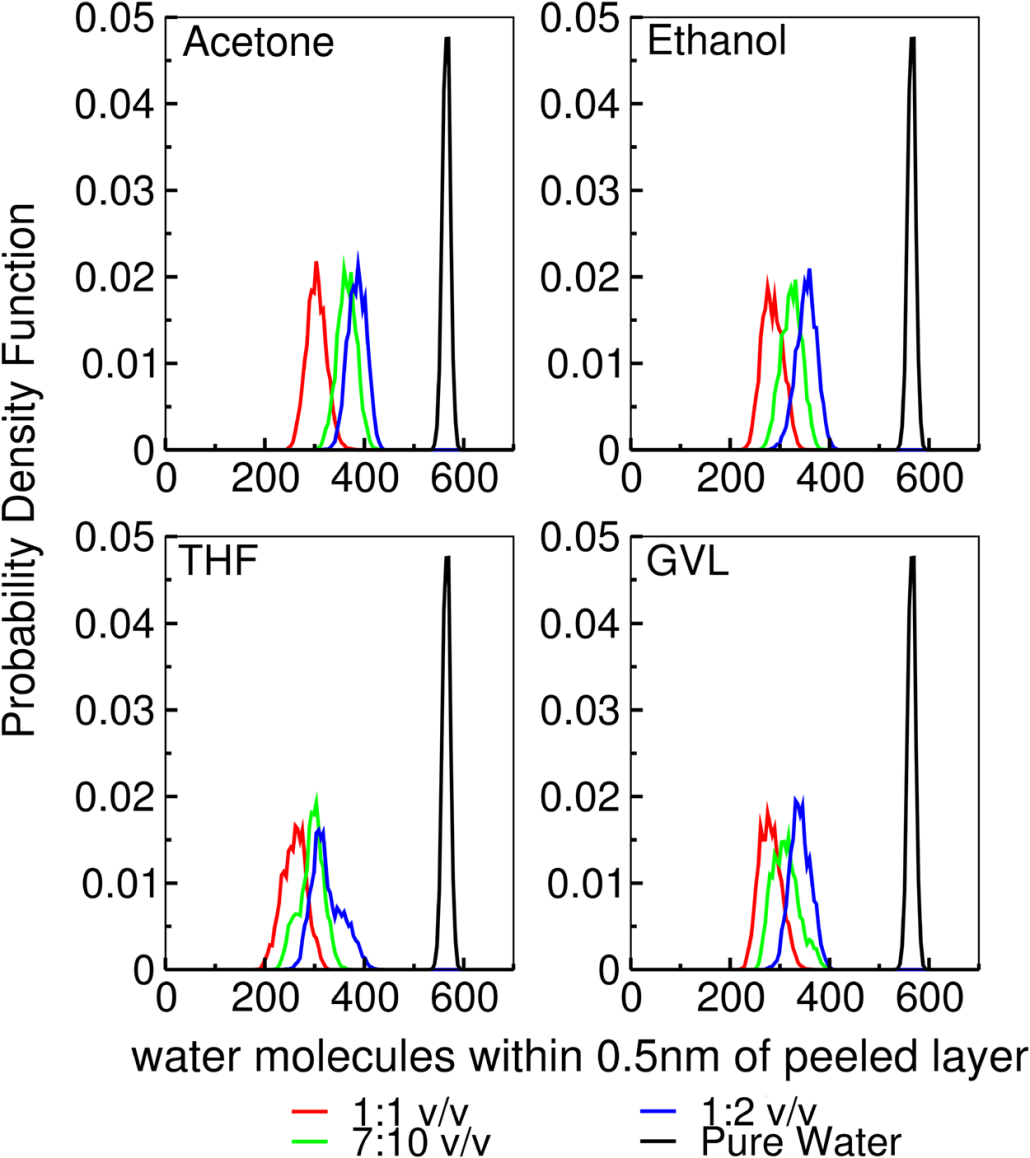
Histograms of the number of water molecules within 0.5nm of the top layer (peeling region) of cellulose.

We first characterise the cellulose-cosolvent interface (Figs 2–5 and ESI Fig. 1) by focusing on the cellulose-water/organosolv radial distribution functions, 2D density profiles projected along the axis perpendicular to the top layer of the cellulose fibre, solvent-cellulose top central strand hydrogen bonding and water lifetimes on cellulose.

Figures 2, 3, and ESI 1, demonstrate that of the four cosolvent systems, the degree of phase separation for the cosolvents follows: THF» GVL > ethanol ≥ acetone, as evidenced by the degree of overlap between the water (dashed) and organosolv (solid) radial distribution profiles and density inhomogeneities in 2D density profiles of the organic solvents. Aside from the nearly concentration-independent phase separation of THF, the other three organic solvents undergo various degrees of patchy demixing at the surface of the cellulose, with GVL having the most pronounced phase separation, followed by ethanol, and finally acetone with a very weak phase separation which is only present at a 1:1 v/v ratio (see ESI Fig. 1). It should be noted;, however, that although a phase separation is evident at the cellulose surface, peaks in the water-cellulose radial distribution profiles below 0.5 nm do indicate that, even when the solvents phase separate, some water remains at the cellulose surface.

Comparing the qualitative ranking of the phase separation of the cosolvent mixtures with the available literature on these water-organosolv cosolvents, we find that the same ranking occurs for their positive deviations from Raoult’s law: THF^41^ and GVL^42^ have relatively large positive deviations from Raoult’s law, followed by a weaker positive deviation for ethanol^43^. Acetone is known to be a negative deviate from Raoult’s law, except at high acetone-water ratios^44^, which is interesting as acetone fails to have any substantial phase separation for the ratios of 7:10 v/v and 1:2 v/v, but does separate for 1:1 v/v. This suggests that when the cohesive interactions (water-water and organosolv-organosolv) outweigh the adhesive (water-organosolv) interactions, the hydrophobic cellulose surface provides enough of a perturbation to the mixture equilibrium to locally split the solvent. We can confirm this by computing the interaction virials between the cosolvent components in the presence of cellulose (Table 1). Table 1 clearly indicates that for the GVL-water and THF-water systems the cohesive (same-same) interactions are substantially more negative (attractive) than the adhesive interactions; indeed, the adhesive interactions are positive (repulsive). Further, for both acetone and ethanol the effect is less pronounced, as both adhesive and cohesive interactions are attractive; however, the cohesive attraction is slightly greater than the adhesive, leading to the weak phase separation observed.

**Table 1:** Solvent interaction virials. Note the B_organosol-organsol_ and B_water-water_ are the cohesive interactions, while B_organsol-water_ is the adhesive interaction between the two cosolvent components.

Cosolvent	Volume Ratio	B_organosol-organosol_	B_water-water_	B_organosol-water_
Acetone	1:1	−0.29	−0.42	−0.33
	7:10	−0.38	−0.35	−0.35
	1:2	−0.24	−0.42	−0.28
Ethanol	1:1	−0.33	−0.52	−0.33
	7:10	−0.32	−0.48	−0.27
	1:2	−0.22	−0.53	−0.22
GVL	1:1	−0.61	−0.92	0.10
	7:10	−0.57	−0.75	0.00
	1:2	−0.49	−0.87	0.20
THF	1:1	−2.89	−2.73	2.10
	7:10	−1.86	−1.91	1.27
	1:2	−2.88	−1.96	1.89

Figures 4 and 5 demonstrates that, for the cosolvent systems, the organic solvent components limit the total number of cellulose chain/strand-solvent hydrogen bonds (as evidenced by the reduction of hydrogen-bonding compared to pure water conditions) and the number of water molecules in the cellulose solvation shell. Further, for the cosolvent mixtures, it is interesting to note that there are only weak differences between them in terms of the magnitude of their reductions of hydrogen bonding and number of cellulose solvation shell water-molecules (relative to pure water conditions).

**Figure 6:**
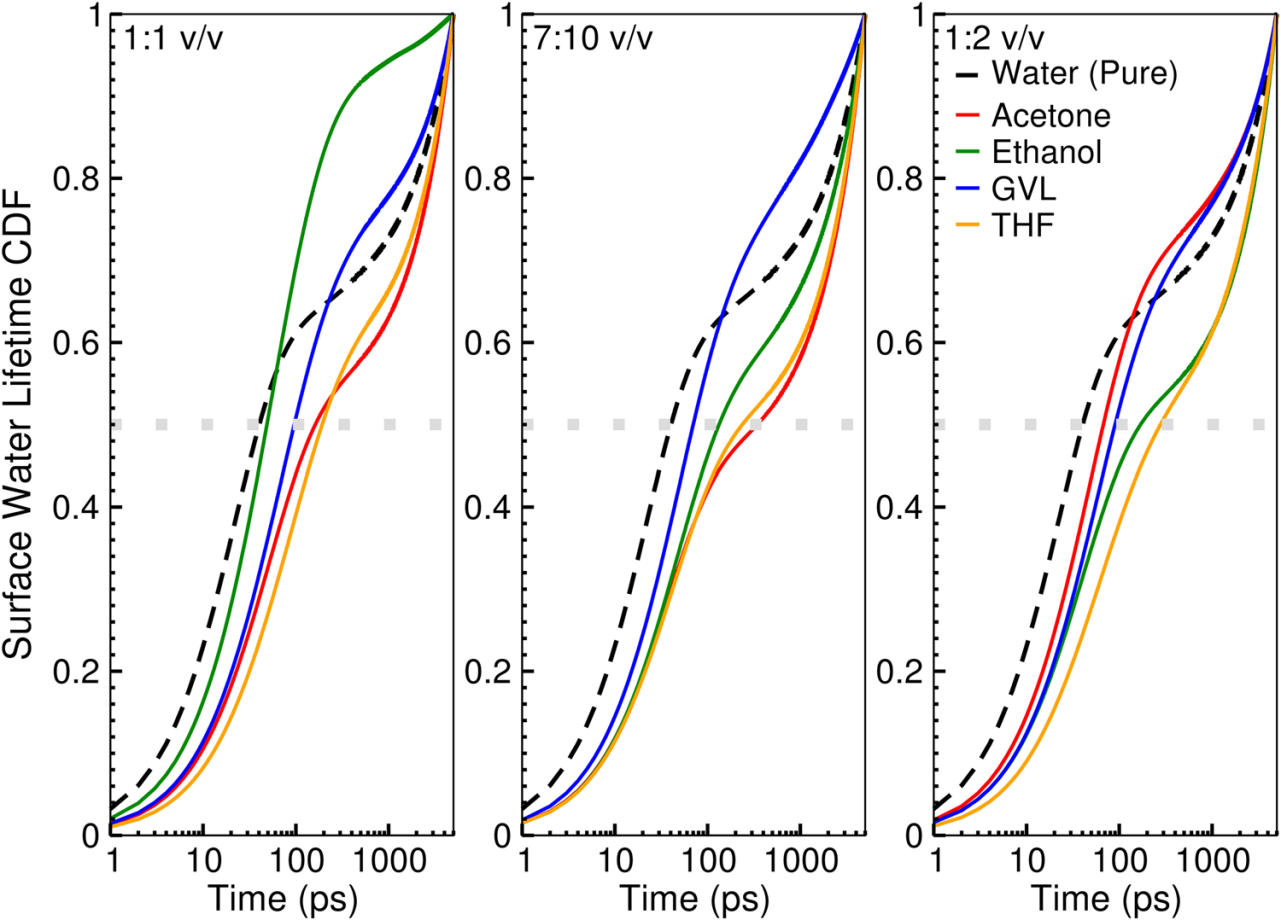
Cellulose water lifetime cumulative distribution functions (CDF). The grey dotted line indicates when the CDF is equal to 0.5.

A major benefit of using molecular dynamics simulations is that temporal relations can be tracked. Figure 6 shows the cumulative distribution functions for water lifetimes on the peeling cellulose surface. Of interest in Fig. 6, is that all cosolvent systems slow surface water compared to pure water conditions, as demonstrated by the median lifetime (time value when the distribution function is equal to 0.5). Furthermore, THF always has the longest lifetimes, while the ranking of GVL, acetone, and ethanol are concentration dependent. An additional point of interest is that, for all concentrations, the GVL distributions cross the water distribution, indicating that although the median lifetime of water at the surface is longer, the distribution is skewed towards shorter lifetimes on average. Considering this data, along with the lack of differences between the cosolvent system in terms of the number of hydrogen-bonds and number of water molecules and their relative differences with pure water conditions, suggests in the case of the THF cosolvent, water near the surface is stabilized. This may explain the experimental breakdown of crystalline cellulose in THF-water cosolvent conditions without the presence of acid, as it is possible that the trapping of water may facilitate THF-water-cellulose reactions, with THF acting as a weak base, to enhance cellulose hydrolysis. Furthermore, it is interesting that this slowing does not occur for GVL cosolvent systems, as it suggests that whatever chemistry that may take place at the surface may not necessarily require stabilized water molecules.

**Figure 7:**
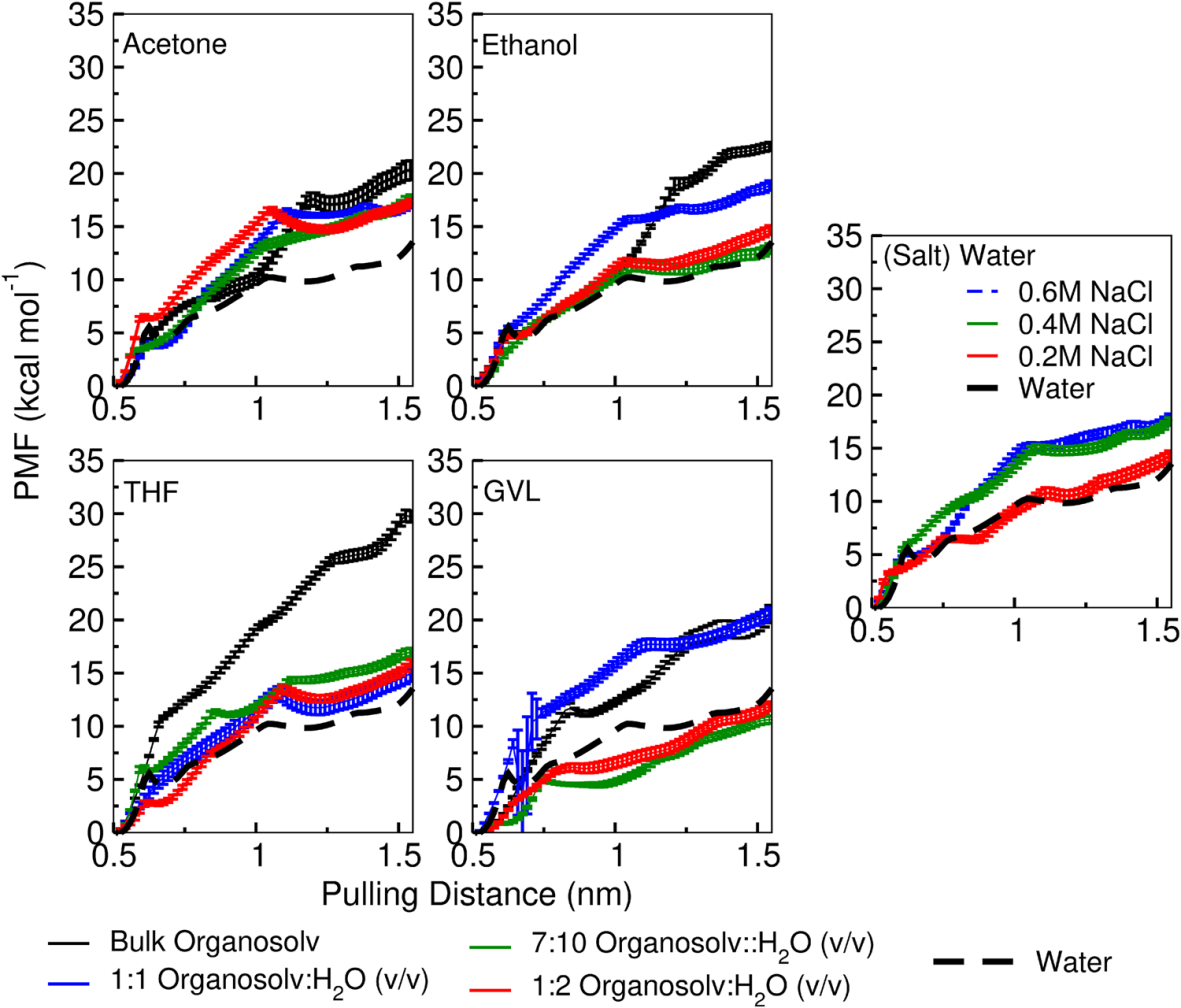
Cellulose dissociation potential of mean force curves. Pulling distance is set such that 0.5nm is the initial z-distance of the end of the pulled chain to the fibre centre of mass. For reference the removal of 1 cellobiose unit (2 glucose monomers) occurs at the pulling distance of roughly 1.05nm (distances are measured from the centre of the fibre). Error-bars are from bootstrapping and are at least the width of the lines. The black curve in the water subfigure is the same as the dashed line in the other four, however, the error bars are shown.

PMF profiles are used to estimate the free energy cost of peeling a central, top layer, chain/strand of cellulose from a cellulose fibre. This permits an estimate of the energy required to perform a stepwise dissociation/decrystallization of cellulose, i.e., dissociation of single glucose and a cellobiose units. To ensure that the utilisation of the chosen reaction coordinate is reasonable, we note that prior computational work on cellulose dissociation (via peeling, as we do here) provides a free energy barrier in water of ~4.4 kcal/mol/glucose unit^38^ and ~7 kcal/mol/cellobiose unit^37,38^. We find that at ~1.05 nm (as shown in the PMF profiles, Fig. 7), dissociation of a single cellobiose unit (two glucose units) in water has an energy cost of ~10 kcal/mol (equivalently ~5 kcal/mol/glucose), and as such has roughly the same free energy cost as previously reported.

For nearly all (co)solvents tested here, the barrier to remove a single unit of cellobiose, as shown in Fig. 7, is either comparable to water or higher. This indicates that, compared to pure water, cosolvent mixtures do not facilitate physical dissociation of cellulose. The exceptions to this trend are found at low GVL to water ratios, as demonstrated by the PMF profiles having either lower or equal energy values to the water PMF profiles (dashed line) at 1.05 nm. Additionally, it is interesting that at a displacement of 1.5 nm from the fibre centre of mass, all tested solvents are again comparable to water or worse, including the previous exception of GVL-water mixtures.

For the cases of the 1:2 and 7:10 v/v GVL-water it is interesting to note that, although the removal of a single cellobiose unit is more favourable than in pure water (red and green profiles are below the dashed black line), the required ~5 kcal/mol is still a substantial free energy barrier. Indeed, given the height of all the barriers noted (greater than 5 kcal/mol) it is clear chain dissociation remains energetically unfavourable under all tested conditions. This suggests that physical (chain) dissociation alone cannot account for the reduction of cellulose under organosolv pretreatments. Instead, the PMF profiles imply that a chemical process would be required to account for any substantial increase in fibre solubilization for the tested cosolvents.

## Conclusions

The present simulation work suggests that organosolv-water cosolvent mixtures have significantly variable phase separation behaviour at the cosolvent-cellulose interface. THF-water, GVL-water, (to a lesser extent) ethanol- and acetone-water cosolvents are all found to demix on the cellulose surface, with the extent of their demixing predictable from their degree of deviation from Raoult’s law. However, the liquid-liquid phase separation of organosolv-water cosolvent mixtures does not directly enhance physical dissociation of cellulose. As both GVL-water and THF-water are known (experimentally) to strongly facilitate cellulose deconstruction, it can be inferred that these two solvents are likely to enhance some type of chemical modifications at the surface, such as bond cleavage. Furthermore, despite their similarities in the chemical structure and the phase separation at the interface, GVL- and THF-water have vastly different influences on water near the cellulose surface, which may result in these two solvent systems having different chemical mechanisms for cellulose-breakdown. This may prove to be a valuable insight as future work will likely focus on the development of catalysts to improve upon the disassembly of cellulose already observed in these water-organosolv cosolvents.

## Electronic supplementary material

Supplementary Information
