# Supplementary material for: Organosolv-Water Cosolvent Phase Separation on Cellulose and its Influence on the Physical Deconstruction of Cellulose: A Molecular Dynamics Analysis
Source: Sci Rep. 2017 Nov 3;7:14494. doi: 10.1038/s41598-017-15048-7 (PMC5670135; doi:10.1038/s41598-017-15048-7)
Supplement: Supplementary file 1 — Supplementary Information [file 41598_2017_15048_MOESM1_ESM.doc]

Electronic Supporting Information for: Organosolv-Water Cosolvent Phase Separation on Cellulose and its Influence on the Physical Deconstruction of Cellulose: A Molecular Dynamics Analysis

Micholas Dean Smith^a, b^, Xiaolin Cheng^a, b, c^, Loukas Petridis^a, b, c^ , Barmak Mostofian^a, b^ and Jeremy C. Smith^a,b*^

Author Affiliations:

a. Center for Molecular Biophysics, University of Tennessee/Oak Ridge National Laboratory, Oak Ridge, TN, 37830 USA

b. Department of Biochemistry and Cellular and Molecular Biology, University of Tennessee, Knoxville, TN 37996

c. Oak Ridge National Laboratory, Oak Ridge, TN 37830 USA

*Corresponding Author: Jeremy C. Smith

Center for Molecular Biophysics

University of Tennessee/Oak Ridge National Laboratory

Oak Ridge, TN, 37830 USA

Phone: 1-865-574-9635/591-4805

Fax: 1-865-576-7651

Email: [smithjc@ornl.gov](mailto:smithjc@ornl.gov)

Organization and layout of this section

This section contains a full display of the 2D density profiles of the organic components of the cosolvent systems at all three cosolvent concentrations. This figure is provided with no additional discussion (aside from figure captions).


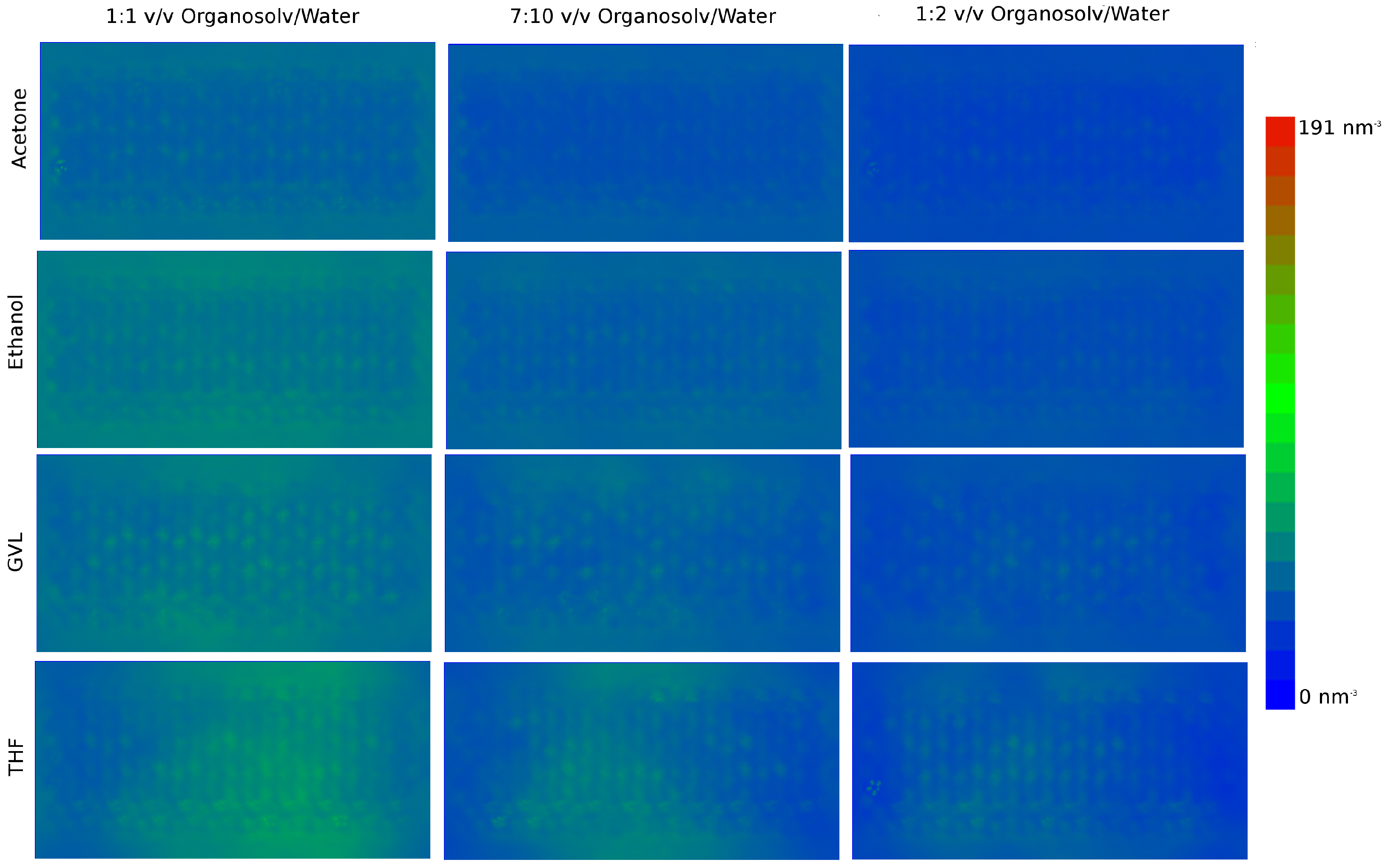


ESI Figure 1) 2D Density Profiles of organosolvs at 1:1 v/v, 7:10 v/v, and 1:2 v/v ratio on the cellulose surface. A) Acetone, B) Ethanol, C) THF, & D) GVL.
